# Clozapine: Improvement of Negative Symptoms of Schizophrenia

**DOI:** 10.7759/cureus.1973

**Published:** 2017-12-20

**Authors:** Afaque H Khan, Samina Zaidi

**Affiliations:** 1 Psychiatry, Heartland Behavioral Healthcare, Department of Mental Health and Addiction, State of Ohio, Northeast Ohio Medical University

**Keywords:** treatment-resistant schizophrenia, clozapine, negative symptoms of schizophrenia

## Abstract

We report five cases of treatment-resistant schizophrenia that presented with prominent negative and positive symptoms. They fulfill the criteria of diagnosis of schizophrenia according to the Diagnostic and Statistical Manual of Mental Disorders, fifth edition (DSM–5). They showed lack of response despite receiving multiple trials of first and second generation psychotropic agents. We decided to give these patients a trial of clozapine to improve their negative and positive symptoms as well as their quality of life. The patients showed various responses to the treatment. Two patients had a robust response to clozapine, two had a moderate response, and treatment was discontinued for one patient due to a side effect of eosinophilia with clozapine, with an eosinophil count increased to 40,000/mm3 (40%).

Clozapine has been established to be more beneficial than conventional antipsychotics in patients with treatment-resistant schizophrenia and apparently more useful, based on existing evidence, in managing the negative symptoms of schizophrenia. We suspect that the improvement in negative symptoms will be associated with an improvement in positive symptoms as well as the compound has a direct action on neuronal pathways responsible for the negative symptoms. Five treatment-refractory schizophrenic patients were given a trial of clozapine for 24 weeks and observed. Overall, two patients showed modest improvement in psychotic and negative symptoms including insight and judgment improvement in disorganization. Two patients demonstrated robust response with significant improvement in negative symptoms including insight, judgment, affect, avolition, and disorganization and also an improvement in psychotic symptoms. We monitored complete blood chemistry (CBC) including the absolute neutrophil count on a weekly basis. Before initiation of the treatment, four patients had all the routine labs performed including glycated hemoglobin (HbA1C), thyroid panel, CBC, comprehensive metabolic panel and fasting lipid profile; all were within normal reference ranges. One patient had type 2 diabetes mellitus (DM) for the past eight years, and his HbA1c was 6.7; that remained stable through the treatment. We managed the symptoms of his type 2 DM with oral hypoglycemic agents and long-acting insulin to control blood sugar and performed a yearly urine analysis for proteinuria to monitor organ damage. His other routine labs were within normal range.

## Introduction

We premise that a fraction of the treatment with clozapine resulting in an improvement in negative symptoms would be linked to an improvement in positive symptoms including hallucination, delusions, and disorganized thought processes. The remainder of its actions would result from its direct action on mesocortical tracts responsible for the negative symptoms [1]. The negative symptoms all improved with clozapine treatment: affective flattening, anhedonia, avolition, apathy, and alogia [2]. Clozapine belongs to a class of second-generation antipsychotics and has been proven to be efficacious with treatment-resistant schizophrenia, acute psychotic disorder, bipolar disorder or mood disorder, drug-induced psychosis, and borderline personality disorder. Clozapine is known to have less adverse effects than those more evident with the first-generation psychotropic medications such as tardive dyskinesia, extrapyramidal side effects, and more anticholinergics. These are disabling, debilitating, embarrassing, and worsen the patient's quality of life.

Recent literature also points out that clozapine is an effective treatment for negative symptoms and positive symptoms of schizophrenia. As we might be aware, negative symptoms such as flat affect, lack of insight and judgment, anhedonia, avolition, poverty of speech and asociality are difficult to manage and are traditionally unresponsive to conventional antipsychotic medications. The presence of negative symptoms in the long term adversely affect the quality of living and deteriorates daily functioning. There is strong evidence that clozapine is also extremely effective in decreasing suicidality, hostility, aggression, and agitation. Schizophrenia is known to associate with self-harm and suicidality. Clozapine, as recent data suggests, significantly decreases suicidal ideations and all-cause mortality in patients with schizophrenia [3]. Several case reports also postulate that improvement in negative symptoms in conjunction with the improvement of positive symptoms is partially linked with its mode of action at the neuronal pathways and pathophysiology involved in schizophrenia [2].

Clozapine is considered a synthetic dibenzodiazepine derivative atypical antipsychotic. Clozapine inhibits several neurotransmitter receptors in the brain: dopamine type 4, serotonin type 2, norepinephrine, acetylcholine, and histamine receptors. Unlike typical antipsychotic agents, it is a blocker of dopamine type 2 receptors. It is known to relieve symptoms of schizophrenia such as hallucinations, delusions, and dementia. Clozapine is an antipsychotic agent that belongs to a class of benzisoxazole derivatives. Clozapine is a selective monoaminergic antagonist with high affinity for the serotonin type 2 (5HT2), dopamine type 2 (D2), alpha-1 and alpha-2 adrenergic, and H1 histaminergic receptors. Clozapine also has antagonistic action on other receptors but with lower potency. Antagonism and relative affinity for other receptors explain other therapeutic and side effects of clozapine. For instance, interaction with the muscarinic M1-5 receptors may explain its anticholinergic effects. Clozapine's antagonist action on histamine H1 receptors explains the somnolence. Agranulocytosis is a main side effect noted with treatment with clozapine and a key reason for discontinuation of treatment. Clozapine's antagonism of alpha-1 adrenergic receptors causes orthostatic hypotension. The side effect of excessive salivation in patients after treatment with clozapine has also been reported in the literature [4].

## Case presentation

We document the case series of five patients who have an extensive history of schizophrenia and fulfill the criteria of diagnosis as defined by the Diagnostic and Statistical Manual of Mental Disorders, fifth edition (DSM-5). They have been inpatients for over a decade, and they presented with prominent negative (i.e., agitation, lack of insight and judgment, irritability) and positive (i.e., hallucination and delusions) symptoms of illness. They showed no response to treatment despite multiple trials of several typical and atypical psychotropic medications including haloperidol, fluphenazine, chlorpromazine, ziprasidone, aripiprazole, paliperidone, and perphenazine and mood stabilizers such as valproic acid and lithium. We had little choice but to utilize clozapine to ameliorate these symptoms. We observed them for 12 weeks with weekly monitoring, complete blood chemistry, and weights. Thyroid panel, HbA1C and comprehensive metabolic panel were in normal reference ranges prior to the treatment with clozapine. We titrated the doses as recommended by the US Food and Drug Administration and increased doses as they were tolerated. The highest dose given ranged between 300 mg to 800 mg per day. Three patients were stabilized on 500 mg or less per day. Current maintenance doses range from 150 mg per day to 700 mg per day. Patients Number 1 and 2 demonstrated a robust response and significant improvement in negative symptoms. They showed improved insight and judgment and less agitation and irritability. They showed improvement in their skills of everyday living. Most notably, they demonstrated improved social interaction and interest by participating in therapeutic groups and outdoor activities as a part of their levels privileges. They also appeared to have an improvement with psychotic symptoms including delusions of paranoia and grandeur, auditory hallucination, and improved thought process with no disorganization and derailment. Their speech was appropriate, coherent, and relevant. The schizophrenia was in partial remission.

Patients Number 3 and 4 exhibited a moderate response. They have shown significant improvement in negative symptoms. However, there was only mild improvement in their psychotic or positive symptoms. Unfortunately, we discontinued the treatment of patient Number 5 with clozapine after the patient developed asymptomatic eosinophilia, with eosinophils increased to 40,000/mm3. We halted the treatment to prevent the risk of organ damage. The patient was diagnosed with an allergic reaction to clozapine [5]. We consulted the hematology and oncology departments for further evaluation. They ruled out any malignancy. The patient’s eosinophil count started trending low and became normal within seven weeks after halting treatment of clozapine. Patients Number 2 and 3 had a clozapine-induced unwanted weight gain of 10 lbs after 16 weeks of treatment. It was troubling, discouraging, and bothersome for the patient, particularly those in the younger population. We started strict dietary and lifestyle  modification in addition to a trial of Glucophage (metformin) at a dose of 500 mg every morning to prevent further weight gain and the development of comorbidities including type 2 diabetes mellitus, coronary artery disease, and metabolic syndrome. Patients Number 3 and 4 had clozapine-induced side effects of sedation/somnolence and fatigue that were addressed by administering higher doses at night and decreasing the dose [6].

## Discussion

As we all might be aware, negative symptoms are disabling, resulting in low functioning living and debilitation in most patients with schizophrenia. These symptoms are resistant to treatment. A lack of motivation is responsible for poor daily functioning, which leads to worsening of relationships with family and friends and asociality. There is also decreased personal interest due to influences of anhedonia, apathy, and inattention. An episode with acute psychotic symptoms is the primary reason for hospitalization and requires pharmacologic intervention to ameliorate psychotic or positive symptoms. Pharmacological treatment effectively decreases re-hospitalization. However, it minimally improves patient functionality, and negative symptoms tend to persevere. Schizophrenia is a heterogeneous disorder manifested by positive, negative, cognitive, and mood symptoms. The severity of symptoms in the above categories and response to treatment varies from patient to patient. Whereas positive symptoms include delusions, hallucinations, suspiciousness, disorganized thinking, negative symptoms constitute a lack of normal function. Negative symptoms include blunting of effect, poverty of speech and thought, apathy, anhedonia, asociality, lack of motivation and social interest, and inattention. These symptoms have devastating consequences on patients’ lives and have only a few modest pharmacological choices for their management.

Most antipsychotics are effective for treating positive symptoms due to their easily identifiable presentation. Negative symptoms are difficult to treat due to confusing and difficult-to-recognize presentation. Patients may manifest symptoms in four domains: affective, communication, relational, and motivational. Patients may appear with deficits in facial expression, eye contact, gesticulation, and prosody. The severity of the symptoms varies with the extent of the disease process. In milder forms, there may be mechanical gestures, and the voice lacks a normal pattern. In severe forms, patients may present with complete lack of expression or communication. They may also have monotonous speech and a blank stare in any direction. In mild forms of speech deficit, patients present with brief and short syntax; in the severe form, a patient can be essentially mute. Even if speech is present, it tends to be vague and monotonous with periodic stillness including increased latency either prior or in the midst of response (i.e., thought blocking). Patients  may demonstrate a lack of motivation, poor personal grooming, and hygiene. They may appear to have difficulty following or adjusting their daily routine or work schedule. Due to limited physical activity and avolition, they have minimal desire to participate in group activities and require frequent prompting and encouragement [7].

## Conclusions

As healthcare providers, we all recognize that the negative symptoms of schizophrenia have been regarded as treatment-refractory. However, they can be managed with pharmacological and social interventions that can make a difference in a patient's life. Psychiatrists face major challenges during the treatment of schizophrenia’s negative symptoms such as subtherapeutic response to the treatment, pervasiveness, and continued decline of the patient's  quality of life. We may be able to mitigate these challenges in the management of negative symptoms by adopting an approach of thorough assessment and adequate treatment. There is evidence that negative symptoms respond to pharmacological intervention, particularly with the second generation antipsychotic clozapine, when it occurs in association with psychotic or positive symptoms. We also suggest utilizing psychosocial treatment such as social skill training, rehabilitation, and psychotherapy in conjunction with pharmacological intervention. We must also continue to document and report case reports of successful treatment of negative symptoms. Also, as healthcare providers, researchers, and clinicians, we must yield all our efforts to explore other treatment options and modalities. Negative symptoms will remain relevant as the main predicament in the schizophrenic patient's quality of life.
